# Expression pattern of circadian genes and steroidogenesis-related genes after testosterone stimulation in the human ovary

**DOI:** 10.1186/s13048-016-0264-5

**Published:** 2016-09-10

**Authors:** Minghui Chen, Yanwen Xu, Benyu Miao, Hui Zhao, Lu Luo, Huijuan Shi, Canquan Zhou

**Affiliations:** 1 Reproductive Medicine Center, The First Affiliated Hospital of Sun Yat-sen University, 58 2nd Zhongshan Road, Guangzhou, GD510080 People’s Republic of China; 2 Guangdong Provincial Key Laboratory of Reproductive Medicine, The First Affiliated Hospital of Sun Yat-sen University, 58 2nd Zhongshan Road, Guangzhou, GD510080 People’s Republic of China; 3 Department of Pathology, The First Affiliated Hospital of Sun Yat-sen University, 58 2nd Zhongshan Road, Guangzhou, GD510080 People’s Republic of China; 4 Department of Hepatic Surgery, The Third Affiliated Hospital of Sun Yat-sen University, 600 Tianhe Road, Guangzhou, GD510630 People’s Republic of China

**Keywords:** Circadian rhythm, Testosterone, Granulosa cells, Ovary, Human

## Abstract

**Background:**

Previous studies have shown that circadian genes might be involved in the development of polycystic ovarian syndrome (PCOS). Hyperandrogenism is a hallmark feature of PCOS. However, the effect of hyperandrogenism on circadian gene expression in human granulosa cells is unknown, and the general expression pattern of circadian genes in the human ovary is unclear.

**Methods:**

Expression of the circadian proteins CLOCK and PER2 in human ovaries was observed by immunohistochemistry. The mRNA expression patterns of the circadian genes *CLOCK*, *PER2*, and *BMAL1*, and the steroidogenesis-related genes *STAR*, *CYP11A1*, *HSD3B2*, and *CYP19A1* in cultured human luteinized granulosa cells were analyzed over the course of 48 h after testosterone treatment by quantitative polymerase chain reaction.

**Results:**

Immunostaining of CLOCK and PER2 protein was detected in the granulosa cells of dominant antral follicles but was absent in the primordial, primary, or preantral follicles of human ovaries. After testosterone stimulation, expression of *PER2* showed an oscillating pattern, with two peaks occurring at the 24th and 44th hours; expression of *CLOCK* increased significantly to the peak at the 24th hour, whereas expression of *BMAL1* did not change significantly over time in human luteinized granulosa cells. Among the four steroidogenesis-related genes evaluated, only *STAR* displayed an oscillating expression pattern with two peaks occurring at the 24th and 40th hours after testosterone stimulation.

**Conclusions:**

Circadian genes are expressed in the dominant antral follicles of the human ovary. Oscillating expression of the circadian gene *PER2* can be induced by testosterone in human granulosa cells in vitro. Expression of *STAR* also displayed an oscillating pattern after testosterone stimulation. Our results indicate a potential relationship between the circadian clock and steroidogenesis in the human ovary, and demonstrate the effect of testosterone on circadian gene expression in granulosa cells.

## Background

A circadian clock is a biochemical mechanism that oscillates with a period of 24 h and is coordinated with the day–night cycle. In mammals, light signals perceived by the retina are transmitted via the retino-hypothalamic tract to the suprachiasmatic nuclei (SCN), which contain the master pacemaker for the generation of circadian rhythms [1]. The SCN synchronize countless subsidiary oscillators existing in the peripheral tissues throughout the body [2]. The basis for maintaining the circadian rhythm is a molecular clock consisting of interlocked transcriptional/translational feedback loops. The proteins encoded by the genes circadian locomotor output cycles kaput (*Clock*) and brain and muscle arnt-like protein 1 (*Bmal1*) heterodimerize and promote the rhythmic transcription of the period (*Per1*, *Per2*) and cryptochrome (*Cry1*, *Cry2*) gene families, whereas modified PER–CRY complexes repress the activity of the CLOCK–BMAL1 complex. Over several hours, PER–CRY complexes are degraded, and the CLOCK–BMAL1 complex is eventually released from feedback inhibition [3–6].

Quantitative reverse transcription-polymerase chain reaction (qRT-PCR) analysis revealed that transcripts for the core oscillator elements (*Arntl*, *Clock*, *Per1*, *Per2*, and *Cry1*) were present in the rat ovary [7]. Expression of *Arntl* and *Per2* was detected in the granulosa and theca layers of growing and antral follicles, as well as in the corpora lutea and stromal fibroblasts of the rat ovary [7]. *Per1* and *Per2* mRNAs in the rat ovary display rhythmic oscillation with a 24-h period [8]. Moreover, such rhythmic expression of a circadian gene can be induced in cultured ovarian cells. When cultured without any treatment, no rhythmic pattern in the expression of either *Per1* or *Bmal1* transcripts was observed in chicken granulosa cells; however, both serum shock and luteinizing hormone (LH) treatment could induce a rhythm of both *Per1* and *Bmal1* in these cells [9].

Polycystic ovarian syndrome (PCOS) is the most common endocrine disorder of reproductive-age women. A recent study highlighted the important role of circadian genes in the development of PCOS. This study showed that the level of *BMAL1* expression in granulosa cells in the PCOS group of women was lower than that of the group without PCOS. Estrogen synthesis and aromatase expression were downregulated after *BMAL1* knock-down and, conversely, were upregulated in KGN cells (a granulosa cell line) overexpressing *BMAL1* [10]. Hyperandrogenism is a hallmark feature of PCOS and is strongly implicated in the genesis of the disorder [11]. A high testosterone level reflects a type of androgen excess, which is one of the primary symptoms of PCOS. In the present study, we selected testosterone as a stimulator for cultured human granulosa cells and observed the temporal expression patterns of circadian genes and steroidogenesis-related genes in human luteinized granulosa cells after testosterone stimulation. In addition, we evaluated the distribution of circadian protein expression in human ovaries by immunohistochemistry.

## Methods

### Immunohistochemistry

Paraffin sections of normal ovarian tissue were obtained from five women aged 29–35 years. All women had undergone bilateral salpingo-oophorectomy with or without hysterectomy for a uterine or unilateral ovarian malignant tumor before chemotherapy or radiotherapy. Informed consent was obtained from all patients. After deparaffinization, antigen retrieval, and blocking in normal goat serum (for CLOCK) or bovine serum (for PER2), the slides were incubated overnight at 4 °C in rabbit anti-human CLOCK polyclonal antibody diluted to 20 μg/mL (catalogue no. ab 65033,Abcam) or goat anti-human PER2 polyclonal antibody diluted to 7.5 μg/mL (catalogue no. ab118489, Abcam), respectively. Slides were washed and incubated with biotin-labeled goat anti-rabbit secondary antibody for CLOCK (CWBIO) or bovine anti-goat secondary antibody for PER2 (Santa Cruz Biotechnology) for 30 min, then washed and incubated with horseradish peroxidase-labeled streptavidin for 10 min. The peroxidase–antibody complex was visualized using 3, 3′-diaminobenzidine (CWBIO). Control experiments included samples treated in the same manner but with omission of the primary antibody. The sections were counterstained with hematoxylin.

### Cell culture

For each experiment, human luteinized granulosa cells were obtained from the follicle fluid collected during ovum aspiration of 10 patients undergoing in vitro fertilization. All patients underwent the long protocol of gonadotropin-releasing hormone agonist treatment (1.0 mg tiptorelin acetate; Ipsen Pharma Biotech, France) with human chorionic gonadotropin as the trigger. The follicle fluid was pooled and centrifuged for 5 min at 2500 rpm. Granulosa cells were purified using 50 % Percoll (Sigma) through gradient centrifugation for 10 min at 2000 rpm. Ovarian tissue fragments were then removed from the granulosa cell suspension with a 40-μm cell strainer (Becton-Dickinson). Purified granulosa cells were plated on a 12-well plate (3.0 × 10^5^/well) and cultured in Dulbecco’s modified Eagle medium/Ham’s F12 supplemented with penicillin (100 U/mL), streptomycin (100 μg/mL), and 10 % fetal bovine serum (Gibco) in a 37 °C incubator with 5 % CO_2_. Cells were cultured for 9 days to reach confluence prior to any treatment. Cells were exposed to 100 ng/mL testosterone (Sigma) dissolved in serum-free medium for 2 h, washed with serum-free medium once, and then cultured further in serum-free medium until harvested. Cells in the control group were cultured in serum-free medium without testosterone treatment. Samples were harvested every 4 h starting at the beginning of treatment and continuing until 48 h.

### RNA extraction and quantification

RNA was extracted with High Pure RNA Isolation Kit (Roche) according to the manufacturer’s instructions, and quantitated by 260/280 UV spectrophotometry (NanoDrop ND-1000, Wilmington, DE, USA). Five hundred nanograms of RNA were subjected to reverse transcription with oligo-dT primers using Roche Transcriptor First-strand cDNA Synthesis Kit. The differential expression of target gene mRNA in granulosa cells was quantified using the following TaqMan® Gene Expression Assays (Applied Biosystems): *CLOCK* (Hs00231857_m1), *PER2* (Hs00256143_m1), *BMAL1* (Hs00154147_m1), steroidogenic acute regulatory protein (*STAR*; Hs00986559_g1), 3-β-hydroxysteroid dehydrogenase type II (*HSD3B2*; Hs00605123-m1), cholesterol side-chain cleavage cytochrome P450 (*CYP11A1*; Hs00167984_m1), and cytochrome P450 (*CYP19A1*; Hs00903413_m1). Quantification was accomplished with an Applied Biosystems 7500 real-time RT-PCR machine using TaqMan® Fast Advanced Master Mix (Invitrogen). The relative mRNA levels were calculated using the comparative cycle threshold method, using *ACTB* (Hs99999903_m1; Applied Biosystems) as a reference gene.

### Statistical analysis

Data are presented as the least squares mean ± standard error of the mean from three replicate experiments. Least-squares analysis of variance (ANOVA) implemented by SPSS 13.0 was used to analyze all data. Tukey’s multiple-comparison post-hoc test was used if equal variances were validated, or Dunnett’s T3 post-hoc test was used if equal variances were not assumed, when ANOVA returned a value of *P* ≤ 0.05. *P* ≤ 0.05 was considered a statistically significant difference.

## Results

### Expression of circadian proteins in the human ovary

**Fig. 1:**
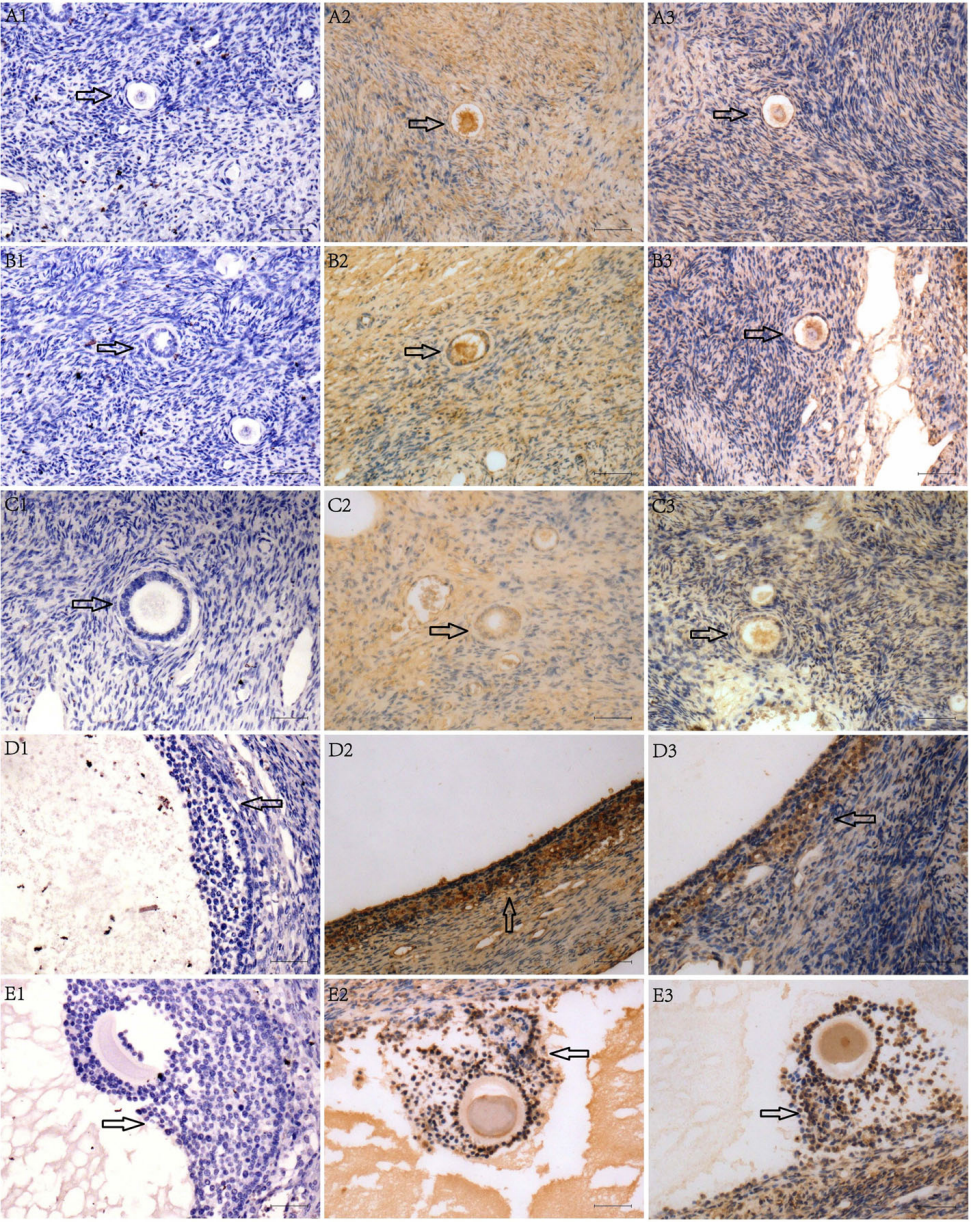
Immunohistochemistry staining of CLOCK and PER2 in paraffin sections of human ovaries. Staining of CLOCK was detected in the cumulus cells and mural granulosa cells, absent in the theca cells of the dominant antral follicles (D2, E2), and present in the interstitial cells, but absent in primordial follicles (A2), primary follicles (B2), and preantral follicles (C2). Staining of PER2 was present in the cumulus cells, mural granulosa cells, weak in the theca cells of dominant antral follicles (D3, E3), and present in the interstitial cells, but absent in the primordial follicles (A3), primary follicles (B3), and preantral follicles (C3). A1 to E1 are negative controls (no primary antibody) of the primordial, primary, preantral, and antral follicles and the cumulus complex, respectively. Bars = 50 μm. Original magnification, ×200

Immunohistochemistry results showed positive expression of CLOCK in the cumulus and mural granulosa cells in dominant antral follicles and in interstitial cells, but no CLOCK expression was observed in the primordial follicles, primary follicles, and preantral follicles, and in the theca cells of antral follicles. Expression of PER2 was observed in the cumulus cells and mural granulosa cells in the dominant antral follicles and in interstitial cells, was weak in the theca cells of the dominant antral follicle, but was absent in the primordial follicle, primary follicles, and preantral follicles (Fig. 1).

### Expression patterns of circadian genes in human granulosa cells stimulated by testosterone

**Fig. 2:**
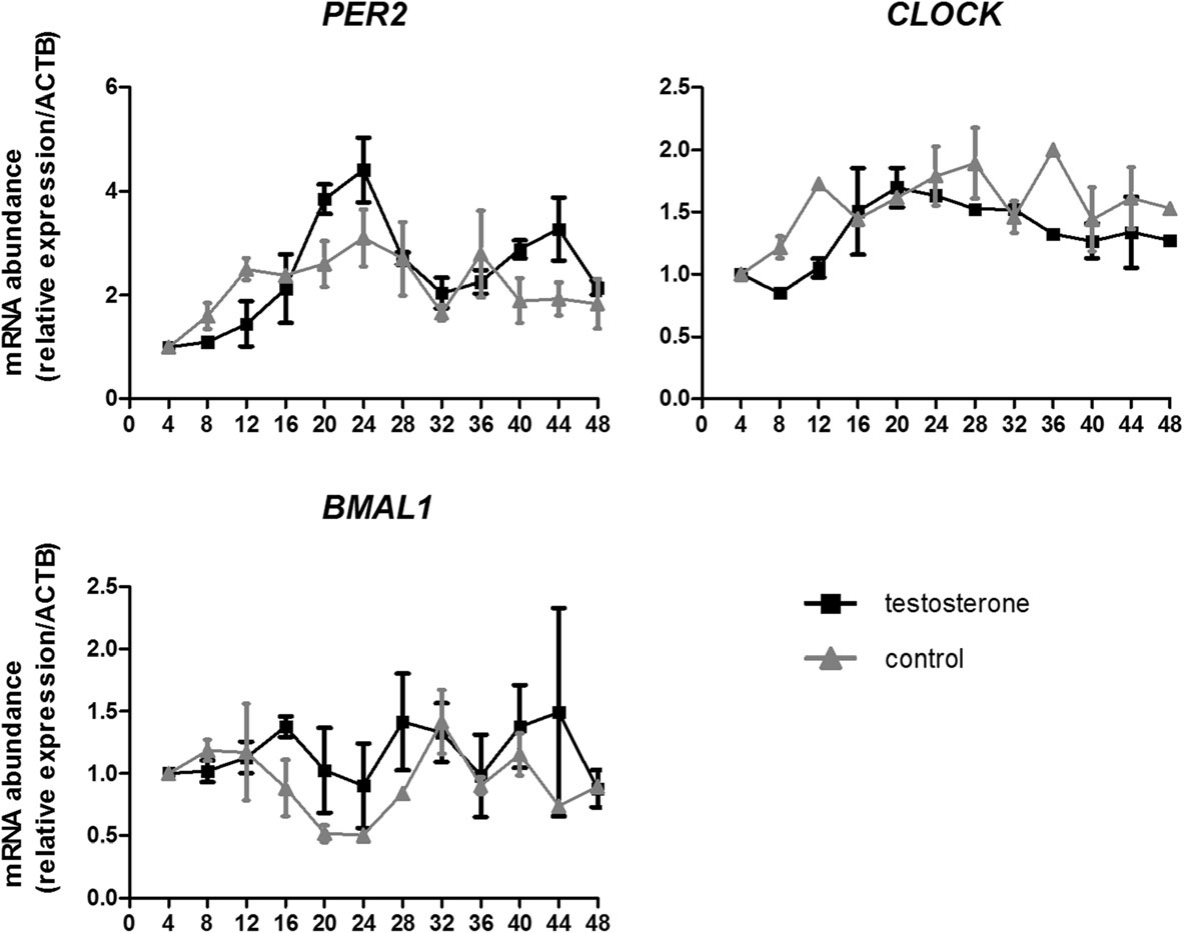
Expression patterns of circadian genes after testosterone treatment in human luteinized granulosa cells. Human luteinized granulosa cells were exposed to 100 ng/mL testosterone dissolved in serum-free medium for 2 h and cells in the control group were cultured in serum-free medium without treatment. Samples were harvested every 4 h from the beginning of treatment for 48 h. Each value represents the mean ± SEM of three independent experiments. Significant statistical differences are shown as below: the testosterone group *PER2* P_4 vs. 24_ = 0.028, P_24 vs. 32_ = 0.041, P_24 vs. 48_ = 0.039, P _4 vs. 44_ = 0.024, *CLOCK* P_4 vs. 24_ = 0.04; the control group *PER2* P_4 vs. 16_ = 0.016, *CLOCK* P_4 vs. 12_ = 0.022, P_4 vs. 16_ = 0.031, P_4 vs. 20_ = 0.006, P_4 vs. 36_ = 0.011

Treatment of testosterone induced oscillations in *PER2* expression, with the first peak and bottom occurring at the 24th and 32nd hours, respectively, and the second peak occurring at the 44th hour (P_4 vs. 24_ = 0.028, P_24 vs. 32_ = 0.041, P_24 vs. 48_ = 0.039, P _4 vs. 44_ = 0.024). Expression of *CLOCK* increased significantly at the 24th hour (P_4 vs. 24_ = 0.04), whereas expression of *BMAL1* did not change significantly after testosterone stimulation. In the control group, after changing to serum-free medium, the expression levels of both *PER2* (P_4 vs. 16_ = 0.016) and *CLOCK* (P_4 vs. 12_ = 0.022, P_4 vs. 16_ = 0.031, P_4 vs. 20_ = 0.006, P_4 vs. 36_ = 0.011) increased significantly; however, they did not display an oscillating pattern. The expression of *BMAL1* did not change significantly over time (Fig. 2).

### Expression patterns of steroidogenesis-related genes in human granulosa cells stimulated by testosterone

**Fig. 3:**
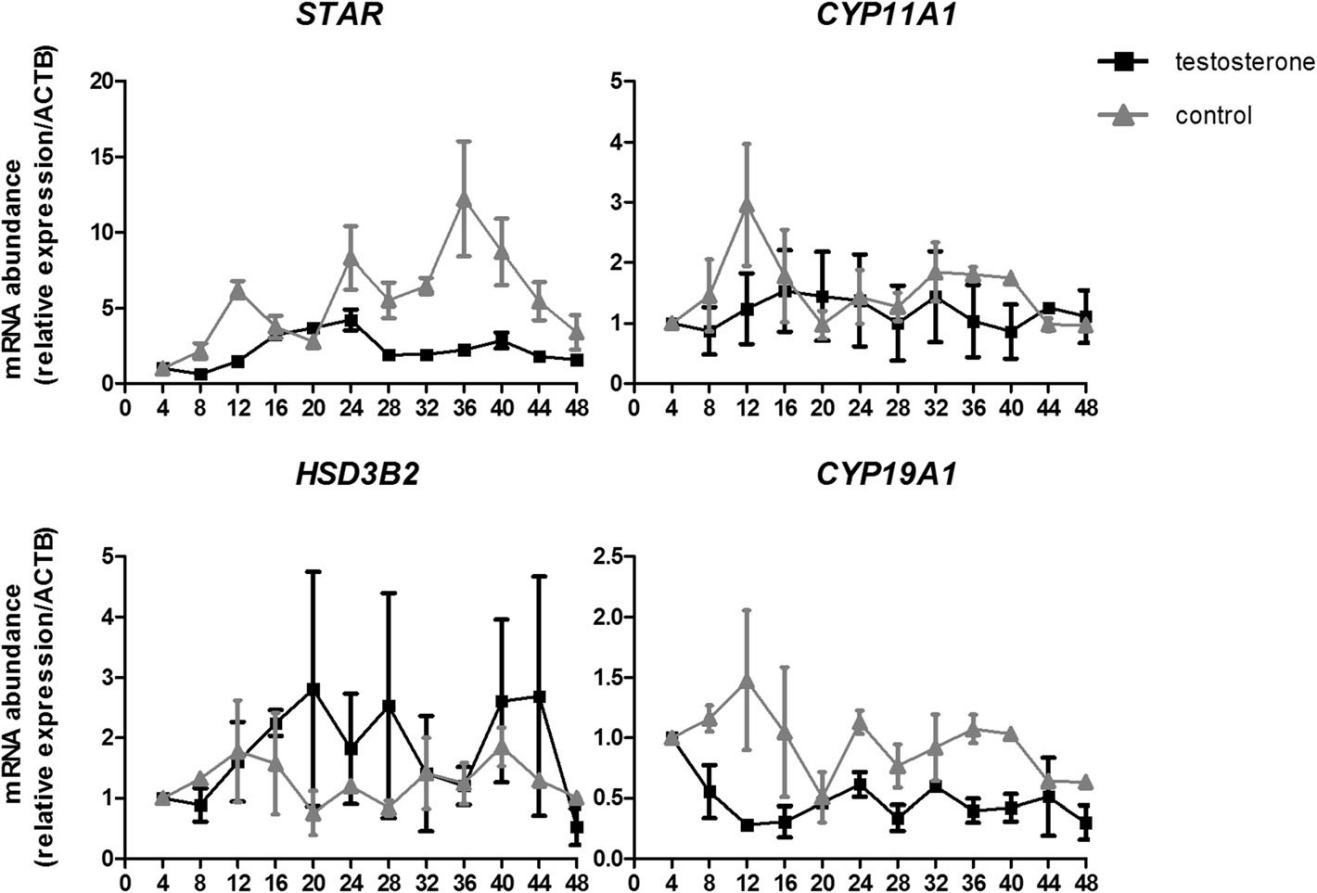
Expression patterns of steroidogenesis-related genes after testosterone treatment in human luteinized granulosa cells. Human luteinized granulosa cells were exposed to 100 ng/mL testosterone dissolved in serum-free medium for 2 h and cells in the control group were cultured in serum-free medium without treatment. Samples were harvested every 4 h from the beginning of treatment for 48 h. Each value represents the mean ± SEM from three independent experiments. Significant statistical differences are shown as below: the testosterone group *STAR* P_4 vs. 24_ < 0.001, P_24 vs. 32_ < 0.001, P_4 vs. 40_ = 0.001), *CYP19A1* P_4 vs. 12_ = 0.003; the control group *STAR* P_4 vs. 24_ = 0.028, P_4 vs. 36_ = 0.001, P_4 vs. 40_ = 0.021

Treatment of testosterone induced oscillations in *STAR* expression, with the first and second peaks occurring at the 24th and 40th hours, respectively (P_4 vs. 24_ < 0.001, P_24 vs. 32_ < 0.001, P_4 vs. 40_ = 0.001). Expression of *CYP19A1* in granulosa cells was significantly repressed at the 12th hour (P_4 vs. 12_ = 0.003), whereas the expression levels of *HSD3B2* and *CYP11A1* did not change significantly after testosterone stimulation. In the control group, changing to serum-free medium significantly stimulated *STAR* expression (P_4 vs. 24_ = 0.028, P_4 vs. 36_ = 0.001, P_4 vs. 40_ = 0.021), but had no significant effects on the expression levels of *CYP11A1*, *HSD3B2*, and *CYP19A1* (Fig. 3).

## Discussion

In this study, we detected the presence of both PER2 and CLOCK proteins in human dominant antral follicles, which were absent in the primordial, primary, and preantral follicles. Moreover, *CLOCK*, *PER2*, and *BMAL1* mRNAs were present in human luteinized granulosa cells. These distribution patterns of circadian genes in the human ovary are similar to those reported for the rat ovary. In the rat ovary, expression of *Per2* and *Bmal1* was detected in growing and antral follicles, as well as in the corpora lutea and stromal fibroblasts [7]. Circadian genes have previously been found to be involved in steroidogenesis in granulosa cells. BMAL1 knock-down can lead to downregulation of estrogen synthesis and aromatase expression; conversely, overexpression of BMAL1 resulted in upregulation of estrogen synthesis and aromatase expression in KGN cells [10]. In addition, inhibition of the expression of Per2 with Per2-specific small interfering RNA stimulated the expression of *Star* in granulosa cells from cows [12]. Both dominant antral follicle and corpora lutea can produce steroids hormone. Expression of circadian genes in dominant antral follicle and corpora lutea of human ovary indicated that expression of circadian gene may also be involved in steroidogenesis in human ovary.

The present study showed that testosterone can induce oscillating expression of *PER2* in human granulosa cells. Although the exact mechanism is unclear, there is some evidence pointing to an association between testosterone and the circadian clock. Testosterone stimulation was shown to promote the association of androgen receptor localized in the plasma membrane with Src kinase, which activated Src kinase [13]. Src-family tyrosine kinases can regulate the expression level of the clock protein Timeless and regulate its function [14]. Moreover, Src activity is involved in the progesterone production of granulosa cells [15]. However, further studies are needed to confirm these relationships.

An experiment on quail in vivo showed that expression of the *Star* gene presented 24-h changes in the largest preovulatory follicle, coinciding with changes in *Per2*. Furthermore, these authors demonstrated that the 5′ flanking region of *Star* contains E-box enhancers that can bind to CLOCK–BMAL1 heterodimers and activate gene transcription [16]. Therefore, these findings indicated that *Star* is a clock-driven gene. Similarly, we found that *STAR* expression showed an oscillating pattern in cultured human granulosa cells after testosterone stimulation, but the pattern did not completely coincide with that of *PER2*. The difference between the results of the present study and those of the quail study may be caused by species-specific differences, the in vivo vs. in vitro study design, or the stimulus used. In addition, we found testosterone can decrease *CYP19A1* expression in human granulosa cells. Our results are in accordance with a recent study which showed that exposure to high level of testosterone could decrease both mRNA and protein levels of aromatase in cultured luteinized granulosa cells isolated from non-PCOS women [17]. However, effect of testosterone on aromatase expression in granulosa cells is different in different species. It has been reported that testosterone stimulates *Cyp19* promoter activity and the expression of *Cyp19* in granulosa cells from immature female rats [18].

A recent study pointed to the important role of circadian genes in the development of PCOS and indicated that circadian clock is likely involved in steroidogenesis in granulosa cells [10]. Our results showed that testosterone can affect circadian gene expression in human granulosa cells. Therefore, hyperandrogenemia can affect circadian gene expression, resulting in the disorder of steroidogenesis in granulosa cells leading to the development of PCOS. However, a limitation of the present study is that we used granulosa cells from hormonally stimulated patients, because it is difficult to obtain granulosa cells from hormonally unstimulated patients. Furthermore, this study represents preliminary research on the expression patterns of circadian genes and their relationship with steroidogenesis in the human ovary. Therefore, further studies are needed to elucidate the molecular mechanism.

## Conclusions

We found that the circadian genes *CLOCK* and *PER2* are expressed in the dominant antral follicles of the human ovary. Testosterone could induce oscillating expression of the circadian gene *PER2* in cultured human granulosa cells. Moreover, expression of *STAR* in human granulosa cells also displayed an oscillating pattern after testosterone stimulation. Our results indicate a potential relationship between the circadian clock and steroidogenesis in the human ovary, and demonstrate the effect of testosterone on circadian gene expression in granulosa cells.

## Abbreviations

BMAL1: Brain and muscle arnt-like protein 1
CLOCK: Circadian locomotor output cycles kaput
CYP11A1: Cholesterol side-chain cleavage cytochrome P450
CYP19A1: Cytochrome P450
HSD3B2: 3-β-hydroxysteroid dehydrogenase type II
PER: Period
qRT-PCR: Quantitative reverse transcription-polymerase chain reaction
STAR: Steroidal acute regulatory protein
